# Analysis of the Optimum Performance for Polymer and Polymer–Nanocomposite-modified Asphalt by Using Multicriteria Decision Analysis

**DOI:** 10.3390/polym16223128

**Published:** 2024-11-09

**Authors:** Mustafa Alas, Shaban Ismael Albrka, Ahmed Eltwati, Ahmed Suliman B. Ali, Allam Musbah Al Allam

**Affiliations:** 1 Department of Civil Engineering, Faculty of Engineering, Near East University, Via Mersin 10, 99138 Nicosia, North Cyprus, Turkey; 2 Department of Civil and Construction management, College of Engineering, A’Sharqiyah University, P.O. Box 42, Ibra 400, Oman; shabarofking10@gmail.com; 3 Department of Civil Engineering, University of Benghazi, Benghazi 12345, Libya; 4 School of Civil Engineering, College of Engineering, Universiti Teknologi MARA, Shah Alam 40450, Selangor, Malaysia; algowel@yahoo.com; 5 Authority of Natural Science Research and Technology, Tripoli 00218, Libya; 6 Libyan Centre for Engineering Research and Information Technology, Bani Walid 00218, Libya; alamalallam8@gmail.com

**Keywords:** rutting and fatigue resistance, nanosilica, ASA, MCDA, TOPSIS, PROMETHEE

## Abstract

The influence of Acrylate Styrene Acrylonitrile (ASA) and ASA/nanosilica (ASA/Si) additives was investigated by using a dynamic shear rheometer (DSR). Firstly, an ASA polymer was blended with the virgin asphalt binder at two different concentrations (3% ASA and 5% ASA). After observing that 5% ASA was the optimum concentration for modification, nanosilica particles were further incorporated into the 5% ASA-modified asphalt binder with two different percentages (5% ASA 3%Si; 5% ASA 5%Si). Frequency sweep tests were conducted across various frequencies at elevated temperatures. The experimental outcomes were analyzed using master curves, rutting, and fatigue resistance parameter plots. Additionally, to provide a more holistic analysis, two different multicriteria decision analysis (MCDA) techniques, namely the Preference Ranking Organization Method for Enrichment Evaluations (PROMETHEE) and the Technique for the Order of Preference by a Similarity to Ideal Solution (TOPSIS), were conducted to identify the best-performing asphalt binder by considering three different parameters: workability, performance under different conditions, and cost. The frequency sweep tests showed that the 5% ASA 5%Si asphalt worked best in terms of resistance to rutting. On the other hand, the virgin binder performed better than all modified binders when it failed to resist fatigue. On the other hand, the PROMETHEE analysis identified the 5% ASA-modified asphalt binder as the optimal choice, while the TOPSIS analysis determined that the 5% ASA 3%Si-modified binder provided the best performance. The differences between the experimental results and the MCDA were due to using more than one evaluation parameter and looking at how well the asphalt binder worked at different temperature ranges at the same time.

## 1. Introduction

In designing asphaltic concrete pavement, it is essential to consider asphalt deterioration over the short- and long-term when the pavement will encounter vehicular loading and diverging environmental conditions [1,2]. Asphalt binder viscoelastic behavior has a considerable influence on the characteristics of the asphalt mixture design, which affects its performance [3,4]. Nevertheless, under severe dynamic loading and extreme weather conditions, a neat asphalt binder is commonly insufficient to provide adequate strength and durability [5]. In environmental conditions where the asphalt pavement is expected to be exposed to intermediate and high temperatures, attention should be given to the asphalt mix design, considering the rutting and fatigue phenomena [6]. Therefore, it is crucial to select an appropriate asphalt binder that aligns with the conditions the asphalt will encounter during its service life to effectively address the aforementioned issues [7]. A method to improve the viscoelastic behavior of asphalt and optimize its performance is to utilize certain additive materials, such as polymers, nanomaterials, and composite materials [8,9,10].

Researchers have already written about the benefits of polymer-modified asphalt binders, such as their better physical, morphological, and rheological properties. These benefits have been confirmed by field evaluations [11]. Therefore, they have been on the market for a long time [12]. However, the two types of polymers that are most frequently used as modifiers in asphalt are plastomers and elastomers. In the same way, the literature acknowledges that elastomers provide promising research results to improve the high-temperature performance characteristics of asphalt as well as improve the low- and intermediate-temperature performance characteristics [13,14]. However, it has also been demonstrated that plastomer modifiers significantly improve high-temperature properties, similar to elastomers, thereby resolving the rutting failure. However, there is still little research that focuses on improvements in asphalt cement performance at intermediate- and low-temperature conditions [15,16].

Plastomeric polymers are commonly formed from the copolymers of polyethylene and polyesters. Plastomers enhance the performance of asphalt binders at elevated temperatures by adding stiffness and plasticity to asphalt features [17,18]. Among the well-recognized types of plastomers, there are polyethylene (PE), polypropylene (PP), and ethylene–vinyl acetate (EVA) [19,20,21]. Another type of polymer used to modify asphalt is the elastomeric polymers from the family of copolymers of polystyrene and polybutadiene, which are also called Styrene–Butadiene (SB) or Styrene–Butadiene–Styrene (SBS). SB and SBS constitute a percentage of more than 75% of the polymers used in asphalt modifications [22,23]. In elastomeric polymers, the presence of polystyrene end-blocks increases the stiffness of the asphalt, while the formation of polybutadiene mid-blocks is responsible for the better elastic properties [24,25]. Aside from SBS, many other thermoplastic elastomers have been used to change asphalt, including Acrylete–Styrene–Acrylonotrile (ASA) and natural rubbers (NRs), which have had similar good results to SBS-modified asphalt binder [26,27,28].

In the current research, special attention has been paid to ASA polymers due to their versatile properties that help improve a number of important features of asphalt. Previously, Hamedi et al. [29] evaluated the influence of ASA-modified HMA fatigue resistance. Their study showed that resistance to fatigue for the asphalt mixtures was significantly enhanced. Another study conducted by Ali et al. [30] also reported that the addition of up to 4% ASA significantly improved the fatigue and rutting resistance for the HMA. Similar experimental outcomes were achieved by Mubariki [31], who additionally conducted morphological analysis on the asphalt binder microstructure. Their findings indicated that a significant enhancement in the rheological properties of asphalt can be achieved with ASA modifications; however, beyond a threshold concentration of 5%, the addition of ASA in the asphalt matrix can lead to a reduction in performance properties due to phase separation and agglomeration, which is also an acknowledged limitation for the commonly used elastomeric polymers in asphalt modifications [32,33].

In the search for enhancing the performance of asphalt binders and mixtures and mitigating the drawbacks of polymer-modified bitumen, nanomaterials and polymer and nanocomposite modifiers have been investigated extensively in recent decades [34,35,36]. Recent research has demonstrated that nanomaterials can be implemented directly as an additive or as a reinforced additive to polymers to enhance the asphalt binder properties further [37,38,39]. The unique qualities of nanomaterials, such as their small particle size, high surface-area-to-volume ratio, and strong dispersion ability in the asphalt matrix make them a possible solution to major deformations, such as rutting and thermal fatigue [40]. Nanomaterials commonly researched in the literature include nano iron, nano clay, and carbon nano tubes [41,42,43,44,45,46]. Nanosilica has been one of the mostly utilized nanomaterials as an additive to asphalt cement. A number of studies have shown that nanosilica, which is also the target modifier used in the current study, can enhance the physical characteristics and viscoelastic behavior of asphalt in addition to its cost-effectiveness [47,48]. According to research carried out by Yao et al. [49], the depth of rutting for HMA can be minimized by up to 50% by adding small amounts of nanosilica in the asphalt blend. Also, Kamboozia et al. [50] found that adding nanosilica to asphalt improves its fatigue resistance, making it up to 37% better than asphalt binder that has not been added to. There have been a significant number of studies found in the literature in which nanosilica was used as a secondary additive in a polymer–nanocomposite matrix. It has been reported in the literature that adding nanosilica to commonly used polymers in asphalt modification, such as SBS, PE, and PP, leads to a significant recovery of their mostly acknowledged drawbacks [51,52].

Several methods that are generally adopted by researchers to analyze the performance of asphalt binder include isochronal plots, master curves, Multiple Stress Creep Recovery (MSCR) plots, rutting resistance, and fatigue resistance plots [53,54]. While these techniques are useful tools, they do not provide a comprehensive understanding of material performance, as the optimal method for determining a well-performing asphalt binder is to conduct an evaluation that considers multiple criteria under all conditions. On this basis, multicriteria decision analysis (MCDA) can be considered a useful tool to conduct such an evaluation. MCDA is a method that enables engineers and authorities to weigh and balance the significance of various criteria and objectives when making a decision [55]. MCDA can be used to assess a variety of choices or substitutes according to how well they perform in relation to various criteria [56].

PROMETHEE and TOPSIS were among the top MCDA techniques that were utilized to evaluate the performance of asphalt binder [57]. Villegas et al. [58] utilized PROMETHEE to determine the optimum performing asphalt cement to be used in asphalt mixtures. Another study conducted by Pamukovic et al. [59] used TOPSIS and focused on the cracking, fatigue, and rutting resistance parameters used to evaluate the optimum characteristics of asphalt binder.

The present study aims to employ the PROMETHEE and TOPSIS methodologies to determine the optimal performance of asphalt binders by considering multiple performance factors. The primary goal of utilizing MCDA was to analyze the experimental results through a comprehensive approach, enabling the identification of the most suitable asphalt binder for specific applications. This research is significant as it provides a robust decision-making framework for selecting asphalt materials, addressing the gap in current methodologies, which often rely on single-factor analysis. By integrating multiple performance criteria, this study contributes to more accurate and reliable material selection for the design of pavement.

## 2. Materials and Methods

The virgin asphalt binder that was also used as the base asphalt for the polymer and polymer–nanocomposite-modified asphalt binders was 80/100 penetration grade and was obtained from Petronas Petroleum/Malaysia. The polymer used in the current study was Acrylate–Styrene–Acrylonitrile (ASA) and was obtained from a company in China called Shijiazhuang Changhang Import & Export Trading, China. Nanosilica was incorporated into the ASA polymer as a secondary additive to form a polymer nanocomposite. Nanosilica was also obtained from the same company mentioned above. The physical specifications of ASA and nanosilica used in this study were provided in Table 1.

Five different asphalt samples, of which two were polymer-modified and two were polymer–nanocomposite-modified asphalt, and a control sample, were prepared. The polymer modification procedure involved the blending of 3% and 5% ASA based on the weight of the virgin asphalt. The polymer–nanocomposite-modified samples were first treated with a 5% concentration of ASA. This was found to be the best amount of polymer-modified binder based on the results of the experiments. Then, the mixing processes were followed by incorporating 3% and 5% Si into the blend to form a polymer nanocomposite. The blending process was conducted at a shear rate of 5000 rpm at 170 °C, and it took a total of 60 min to achieve a homogenous blend, which was acknowledged by observing the softening point of the samples at 20 min intervals during the blending process. For the control sample, only the manual stirring technique was utilized. In this manuscript, the samples are labeled as “Base AC” for the control sample, “3% ASA” and “5% ASA” for the samples that had the polymer changed, and “5% ASA-3% Si” and “5% ASA-5% Si” for the samples that had the polymer nanocomposite added.

### 2.1. Physical Properties

The physical parameters of the asphalt binders were determined through the implementation of standard ASTM testing protocols. To test the physical properties, penetration, softening point, and rotational viscosity tests were utilized. The penetration test enabled us to confirm the penetration grade that was specified by the manufacturer. Additionally, softening point tests have aided in determining the temperature, time, and shear rate of the blending procedures used in the preparation of the asphalt sample. Also, to determine the workability of the prepared blends, a rotational viscosity test was performed by using a Brookfield rotational viscometer and adopting the ASTM D4402 testing procedures.

### 2.2. Frequency Sweep Tests

**Figure 1:**
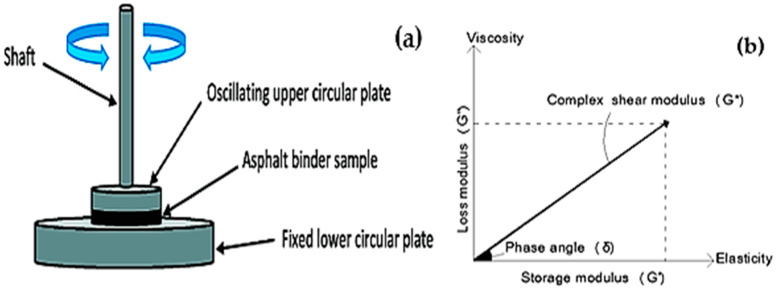
(**a**) Schematic of DSR. (**b**) Representation of G* and δ.

Frequency sweep experiments were performed by a dynamic shear rheometer (DSR) between 10 and 75 °C in increments of 10 °C and at frequencies ranging from 0.159 to 15 Hz (0.159, 0.2, 0.5, 1, 1.592, 2, 5, 10, and 15 Hz) in order to evaluate the rheological characteristics of both the virgin binder and the modified binders. The tests were conducted by applying stress in sinusoidal wave form under strain-controlled conditions. The DSR is equipped with fixed (bottom) and oscillating (top) plates; the oscillations of the top plate at different frequencies create a shearing action on the asphalt samples. A 25 mm diameter plate with a 1 mm gap between the plates was used to test the samples at temperatures higher than 45 °C. A plate with an 8 mm diameter and a 2 mm gap between the plates was used to test the samples below 45 °C. A constant and stable temperature environment was achieved by performing the tests in an automated fluid bath system. The geometry of the plates varied according to the test temperature. The schematic of the experimental setup is illustrated in Figure 1a. 

The test is used to determine the complex modulus (G*) and phase angle (δ) at different temperatures and frequencies in order to evaluate the resilience of the asphalt binders to fatigue and rutting. The results of the frequency sweep test are shown in Figure 1b. Rutting is a significant issue that arises during the construction, transportation, and placement phases of asphaltic concrete. However, fatigue becomes the main concern during the later stages of the lifespan of the asphalt concrete. Based on this fact, the laboratory samples were first subjected to short-term ageing by applying the rolling thin film oven (RTFO) and long-term ageing by using the pressure ageing vessel (PAV). The fatigue resistance measures were assessed after subjecting the samples to ageing methods. No conditioning was performed to the samples that were examined for rutting.

### 2.3. MCDA

MCDA was utilized to assess the optimum concentrations of the modifiers by considering the enhancements in workability, rheological properties, and the increase in cost due to the application of modifiers. The analysis included asphalt samples (Base AC, 3%ASA, 5%ASA, 5%ASA-3%Si, and 5%ASA-5%Si) as alternatives. Lower viscosity for the asphalt was considered better workability since this would indicate lower pumping, mixing, and compaction temperatures during the production and placement of asphalt concrete. Enhancements in rheological properties were accounted by considering the rutting and fatigue resistance parameters at elevated temperatures and different frequencies. Finally, the cost of modification was taken into account by considering the percent increase in the cost after the modification, which was achieved by comparing the baseline cost per ton and the total cost per ton, as expressed in Equation (1) below.

In the MCDA analyses, the high or low values of the above-mentioned parameters can provide advantages and/or disadvantages to an alternative. For instance, in the case of an increase in cost, the minimum value is considered advantageous, while the maximum value is viewed as a disadvantage. Conversely, when considering improvements in rheological properties, maximum improvement is seen as advantageous, whereas minimal development is considered a disadvantage. For this reason, minimum or maximum, as a better approach, was utilized in comparing the alternatives with the above-mentioned criteria.

(1) $$Percentage\;increase=\left(\frac{Total\;cost\;per\;ton-Baseline\;cost\;per\;ton}{Baseline\;cost\;per\;ton}\right)\times 100\%$$

#### 2.3.1. PROMETHEE

Compared to other MCDM methods, PROMETHEE is considered a basic ranking method in terms of both concept and analysis. The calculation procedures for the PROMETHEE need several steps, and they are summarized as below:

Step 1: Identifying the set of potential alternatives and the criteria (j = 1,…, k).

Step 2: Calculating the weight of each criterion (w_j_). This indicates the relative significance of each criterion and signifies that

(2) $$\sum_{j=1}^{k}w_{j}=1$$

Step 3: The decision matrix is normalized between 0 and 1 by using

(3) $$R_{ij}=\frac{\left[X_{ij}-\min\left(X_{ij}\right)\right]}{\left[\max\left(X_{ij}\right)-\min\left(X_{ij}\right)\right]}$$

(i = 1, 2…n and j = 1, 2…m),

The variable X_ij_ represents the evaluation values provided by the decision-makers, where i ranges from 1 to n and j ranges from 1 to the number of criteria m.

Step 4: Determining perturbation with pairwise comparison

d_j_(a, b) = g_j_(a) − g_j_(b) (4)

d_j_ (a, b) indicates the discrepancy among “a” and “b”’ evaluations for each criterion.

Step 5: Defining the preference function

P_j_(a, b) = F_j_ [d_j_ (a, b)], (5)

Function P_j_(a, b) calculates the variance in the evaluations for alternative “a” relative to alternative “b” on each criterion and then scales it to a range from 0 to 1. The defects of the decision-maker are represented by a finite set of functions. On the other hand, the degree of liking increases as the value approaches 1.

Step 6: Computing the multicriteria preference index.

(6) $$\pi \left(a,b\right)=\sum_{j=1}^{k}P(a,b)w_{j}$$

w_j_ > 0 is the weight associated with each criterion, and π (a, b) represents that the degree of “a” is preferred over “b” for all the criteria.

π (a, b) ≈ 0 denotes a slight preference for “a” compared to “b”;

π (a, b) ≈ 1 denotes a high preference for “a” compared to “b”.

Step 7: Obtaining the preference list

This step enables us to obtain a partial or full ranking. PROMETHEE I is often used to obtain partial ranking, while complete ranking is obtained by adding one more phase to the computation using PROMETHEE II. The computation methods remain fixed, except for step 5. In step 5, the decision-maker’s preferences, along with the criterion features, significantly influence the arbitrary selection of the preference functions.

#### 2.3.2. TOPSIS

To determine which solution is the closest to the ideal positive solution and the furthest from the ideal negative solution, the TOPSIS technique is utilized. Knowledge of the relative value of qualities is necessary in TOPSIS, as they play a crucial role in the selection process. The following procedures are implemented in TOPSIS.

Step 1: The equation expressed below is used to normalize the decision matrix:

(7) $$N_{ij}=\frac{x_{ij}}{\surd \sum_{i=1}^{m}{x_{ij}}^{2}}\;\;\;\;\;\;\;\;j\;=\;1,\;2\;\ldots \;n;\;\;\;i\;=\;1,\;2\;\ldots \;m$$

Step 2: Following the acquisition of the normalized data from the previous phase, the decision matrix is multiplied by the corresponding weights by using Equation (8) to produce the weighted decision matrix.

V_ij_ = n_ij_w_j_   j = 1, 2 … n   i = 1, 2 … m (8)

Step 3: The following equations can be used to find the ideal and nadir ideal solutions.

{V_1_+, V_2_+ … V_n_+}{(max_i_V_ij_|jϵk), (min_i_V_ij_|jϵk), |i = 1, 2 … m} (9)

{V_1_−, V_2_− … V_n_−}{(min_i_V_ij_|jϵk), (max_i_V_ij_|jϵk), |i = 1, 2 … m} (10)

where K denotes both the index set of cost criteria and the index set of benefit criteria, respectively.

Step 4: Equations (10) and (11), after computing the distances between the ideal and nadir ideal solutions, are used to determine the two Euclidean alternatives.

(11) $$S_{i}^{+}=\{\sum_{j=i}^{n}({V_{ij-}V_{j}^{+})}^{2}\}\times \;0.5\;\;\;\;\;\;\;\;\;j\;=\;1,\;2\;\ldots \;n;\;\;\;i\;=\;1,\;2\;\ldots \;m$$

(12) $$S_{i}^{-}=\{\sum_{j=i}^{n}({V_{ij-}V_{j}^{-})}^{2}\}\times \;0.5\;\;\;\;\;\;\;\;\;j\;=\;1,\;2\;\ldots \;n;\;\;\;i\;=\;1,\;2\;\ldots \;m$$

Step 5: To determine the relative proximity to the optimal solution, Equation (13) is applied:

(13) $$C_{i}=\frac{{Si}^{-}}{{Si}^{+}+{Si}^{-}}\;\;\;\;\;\;i\;=\;1,\;2\;\ldots \;m\;\;\;\;\;0\;\leq \;Ci\;\leq \;1$$

Higher ranks are represented by greater C_i_ values.

## 3. Results and Discussion

**Figure 2:**
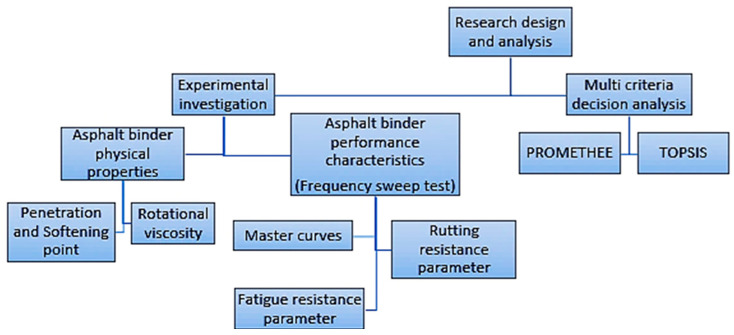
Schematics for the research design and analysis of the results.

The current study split its results into two distinct sections. In the first section, the experimental outcomes were evaluated to determine the physical and rheological properties of neat and modified asphalt binders. The evaluation was particularly focused on assessing the viscoelastic behavior of the test samples. In the second section, the results of the physical and rheological characteristics of asphalt binders were analyzed by two distinct multicriteria decision analysis. TOPSIS and PROMETHEE have been utilized as part of the investigation. The schematic of the investigation was demonstrated in Figure 2.

### 3.1. Asphalt Binder Conventional Properties

**Figure 3:**
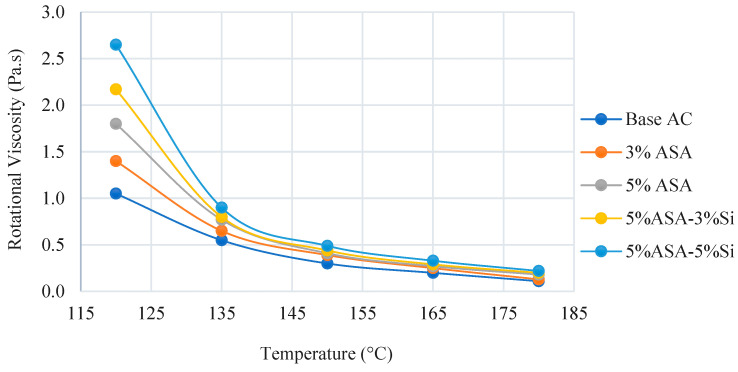
Rotational viscosity of asphalt binders.

The physical attributes of the five distinct asphalt blends are shown in Table 2 below. Furthermore, Figure 3 displays the outcomes of the rotational viscosity tests. The graph is extended from 120 to 180 °C to show how high temperatures affect viscosity and how the modification process affects the virgin asphalt binder.

### 3.2. Rheological Results

#### 3.2.1. Master Curves

**Figure 4:**
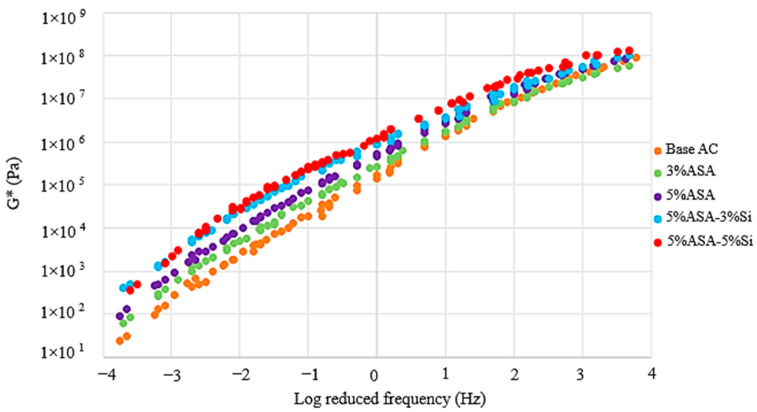
DSR test results for G*.

**Figure 5:**
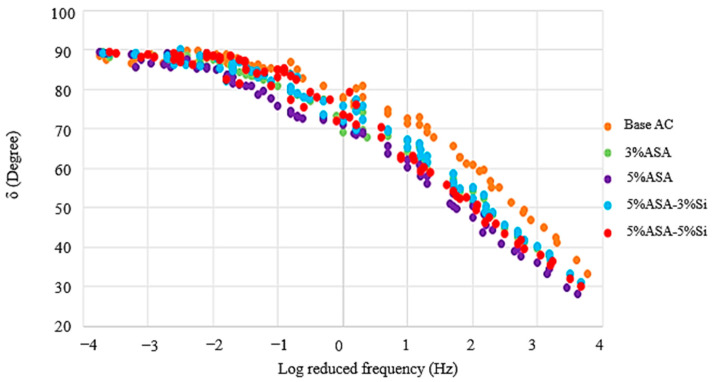
DSR test results for δ.

Figure 4 and Figure 5 were generated utilizing the time-temperature superposition theory. G* represented stiffness, while δ provided insight about the elasticity of the asphalt blends. For an asphalt binder to possess better viscoelasticity, it is desirable that the G* is higher in cases of high temperatures and low frequencies, and the opposite is vice versa. Figure 4 shows that 5% ASA/5% Si achieved the highest G*, followed by 5% ASA/3% Si, with the control sample showing the lowest enhancement in G*. This result demonstrated that the incorporation of nanosilica into the polymer asphalt blend gave the optimum performance for low-frequency and high-temperature environments.

On the other hand, the deductions from Figure 5 illustrated that the best-performing asphalt binder among the alternatives was the control sample in terms of resisting fatigue cracking. On this basis, it would not be incorrect to conclude that the modification process has caused a significant increase in stiffness, resulting in lower elastic properties for the ASA and ASA–nanosilica-modified samples. Notably, adding nanosilica to the polymer asphalt mix barely lowered the performance of the asphalt at normal temperatures. The 5%ASA asphalt binder had the least resistance to fatigue cracking. A possible reason for such a result could be explained by the occurrence of phase separation between the ASA and the asphalt binder, which has also been mentioned in a number of previous studies [27,60].

#### 3.2.2. Parameters of Rutting and Fatigue

**Figure 6:**
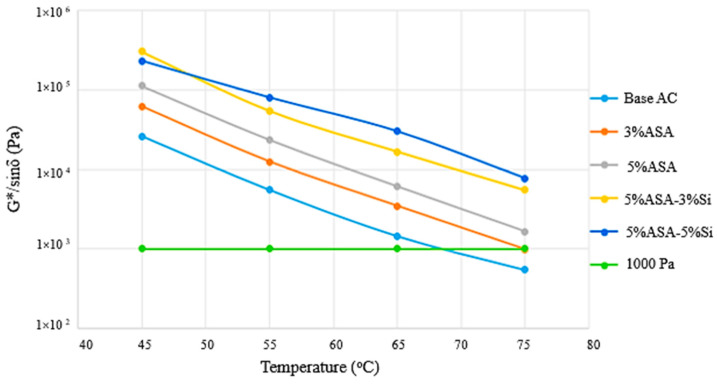
Temperature dependence of the rutting resistance parameter.

Rutting resistance (G*/sinδ) and fatigue resistance (G*.sinδ) parameters were plotted by using the DSR test results. The rutting resistance of unaged asphalt samples was measured at temperatures between 46 °C and 82 °C at a frequency of 1.59 Hz. On the other hand, fatigue resistance was measured for PAV-aged samples between the temperatures 10 and 35 °C at a frequency of 1.59 Hz, as identified in the Superpave specification. Figure 6 illustrates the G*/sinδ for the virgin and modified asphalt cement samples. It was observed that the performance grade of the neat asphalt cement was 64 °C, while G*/sinδ was remarkably enhanced after the modification process.

**Figure 7:**
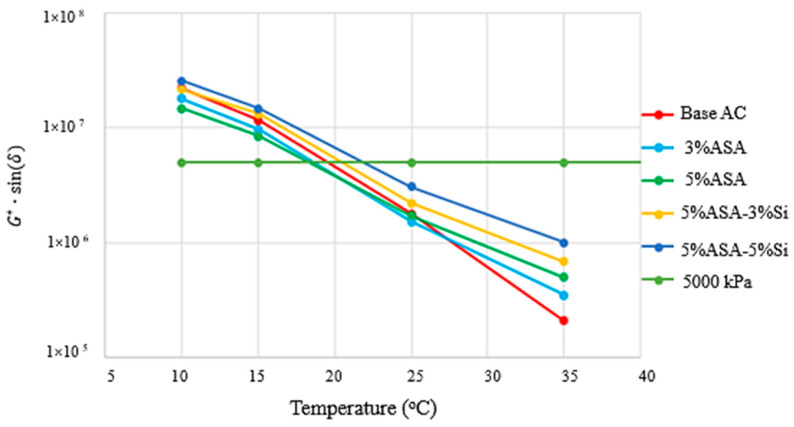
Temperature dependence of the fatigue resistance parameter.

The fatigue resistance parameter was calculated by multiplying the frequency sweep test values of G* and δ. The main difference between the fatigue resistance parameter and the rutting resistance parameter is that the fatigue resistance parameter was determined by using initial short- and long-term ageing operations. The maximum permissible value for asphalt cement’s fatigue resistance is 5000 kPa, according to the Superpave reference. Figure 7 unequivocally shows that the treated asphalt samples’ fatigue resistance measure has significantly decreased. The decrease in the fatigue resistance value was notably greater for the ASA/Si-modified samples. Consequently, it is plausible to assert that the modification technique greatly improved the rutting performance of the asphalt binder; however, the cost of modification appeared to be losing elasticity for the binders, which was also not able to be recovered significantly by the nanosilica addition to the ASA polymer asphalt matrix.

### 3.3. MCDA Results

#### 3.3.1. PROMETHEE

PROMETHEE is an instrument for the assessment and prioritization of a number of alternatives in decision-making based on the pros and cons of each alternative. In PROMETHEE, the available alternatives are compared pairwise and assigned a preference index (Phi+) and a dispreference index (Phi−) for the most preferred and least preferred alternatives, respectively. The net index (Phi) is the result of the differences between the preference indices mentioned above. Based on the Phi value, the alternative options are ranked from top to bottom, with a higher phi value indicating a preferable option.

**Figure 8:**
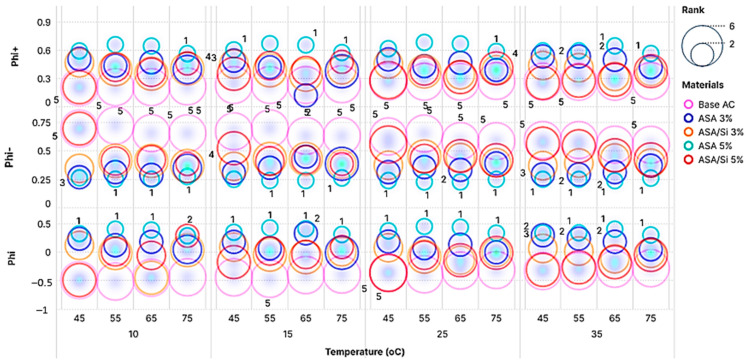
PROMETHEE’s performance and ranking.

The PROMETHEE analysis results are illustrated in Figure 8. Phi+, Phi−, and net Phi values were illustrated on the y-axis, while the range of testing temperatures was represented on the x-axis. The x-axis was divided into four sections representing low-temperature ranges (10 °C, 15 °C, 25 °C, and 35 °C) and on top of the classified low-temperature groups can be seen the high-temperature ranges for each group (45 °C, 55 °C, 65 °C, and 75 °C). The above explanation means that the rank performance for each asphalt binder was given according to its performance at both low- and high-temperature levels.

The ranking was performed according to the net Phi values. As deduced from Figure 8, there was a clear distinction between the 5% ASA and control binder and the other alternative modified asphalt binders. The second-ranked alternative was observed to be 3% ASA in most of the ranking tables, and the worst-ranked alternative was the control sample, which indicated the effectiveness of the modification process. Moreover, the results observed in Figure 8 highlight varying material responses to temperature changes, as evidenced by the distribution of bubble sizes across temperatures. Notably, 5% ASA appears to consistently exhibit larger bubbles across temperatures, suggesting a potentially higher overall ranking. Apart from the regular trend in the analysis results, at certain temperature ranges, it can be observed that second, third, and fourth ranked samples have shifted ranks due to changes in the behavior of asphalt samples at different concentrations at different temperatures and frequencies.

#### 3.3.2. TOPSIS

The purpose of utilizing the TOPSIS analysis was to evaluate the optimum concentration of modified asphalt binder that performs well under all temperature and frequency conditions, as well as taking into account the workability and cost factors. Similarly to the PROMETHEE method, the results were expressed in terms of ranks; however, different from the PROMETHEE method, TOPSIS is based on the geometric distance of each alternative to an ideal (best) and a negative ideal (worst) solution. It ranks alternatives based on their relative closeness to the reference points.

**Figure 9:**
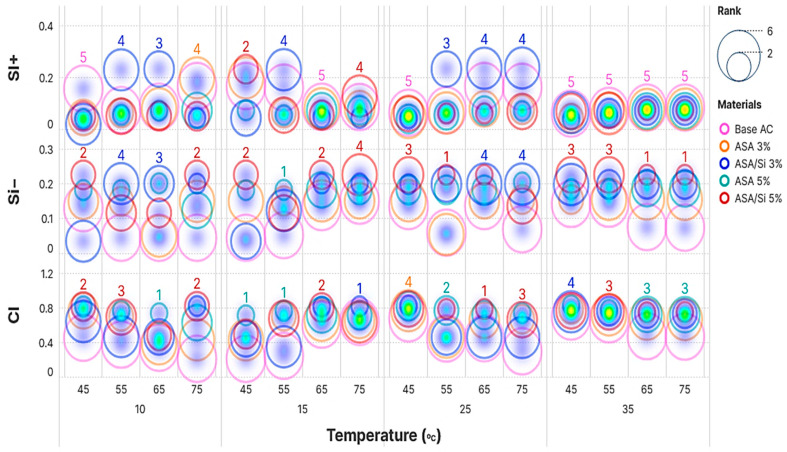
TOPSIS performance and ranking.

The outcomes of the TOPSIS analysis are presented in Figure 9. The asphalt samples being compared were Base AC, 3% ASA, 5% ASA, 5%ASA-3%Si, and 5%ASA-5%Si. The range of temperatures at which the asphalt performance was evaluated were represented by the horizontal axis, while the vertical axis illustrated the Euclidean distance between alternatives and the relative closeness values to selecting the optimal alternative.

Figure 9 depicts a bubble distribution chart illustrating the results of the TOPSIS analysis. Based on the CI values, it was found that the best mix of additives for the asphalt binder was 5%ASA to 3%ASA, especially at higher temperatures. This outcome was reasonable since the polymer–nanocomposite effect is expected to overcome unfavorable issues (rutting) at high temperatures. This has demonstrated that although the workability of the asphalt is reduced and although the associated cost due to the implementation of additives is increased, the improvement in performance outranked the downsides of the modification process. Notably, at lower temperature ranges, it was observed that 5% ASA modified asphalt binder occasionally surpasses the 5%ASA-3%ASA combination in terms of overall ranking. This outcome was also reasonable, as the improvement in performance did not offset the associated increase in the cost of the additives.

It is noteworthy to mention that both PROMETHEE and TOPSIS yielded similar results; however, there have been some discrepancies in the results observed, particularly at high temperature ranges. A possible reason for the differences in the analysis results could be explained by the distinct methodologies and approaches they employ. PROMETHEE uses pairwise comparisons of alternatives based on preference functions to calculate net outranking flows. It focuses on how one alternative performs relative to another, considering both the strength and direction of preference. It generates a complete or partial ranking based on the outranking flows (positive and negative), which represent how much an alternative is preferred over the others. On the other hand, TOPIS adopts a distance-based calculation technique which relies on the geometric distance of each alternative to an ideal (best) and a negative ideal (worst) solution. It ranks alternatives based on their relative closeness to these two reference points.

The techniques employed in the current study, PROMETHEE and TOPSIS, can be beneficial in a variety of situations and for different purposes. TopSIS may be better if there are clear criteria, and if the goal is to find the alternative closest to the optimal solution. Conversely, PROMETHEE may be more suitable if the consideration is the positive and negative aspects of each alternative together, and if the alternatives are ranked based on their overall performance.

## 4. Conclusions

This study was conducted in two parts. In the beginning, the results of the experiments were analyzed using standard methods for showing the viscoelastic properties of the base, polymer, and nanocomposite-modified asphalt binders. In the second part, we carried out MCDA using the TOPSIS and PROMETHEE techniques to assess the experimental outcomes, taking into account improvements in workability and rheological properties, as well as the increased costs associated with the use of modifiers. The following conclusions were drawn from the current study.

- Based on the experimental outcomes from the frequency sweep tests, 5%ASA-5%Si was found to perform superiorly over the base asphalt and the other combinations of modified asphalt binders at high-temperature conditions.
- On the contrary, it was observed that the performance of modified asphalt binders was poor to resist fatigue failure at low temperatures since the modification process yielded an increase in stiffness. For this reason, Base AC was the optimum performing asphalt binder if the low-temperature performance is taken as the only criteria in the evaluation.
- As part of the MCDA, the PROMETHEE analysis results demonstrated that 5%ASA outperformed the other alternative combinations of modified asphalt binders. The primary reason for this was the inclusion of multiple criteria in the analysis, which considered the performance of the asphalt binder across all temperature and frequency ranges.
- The TOPIS analysis results presented similar findings to the PROMETHEE results; however, the optimum performing asphalt binder was found to be the 5%ASA-3%Si combination of modifiers. The small discrepancies between the TOPSIS and PROMETHEE analysis results were justified by differences in the methodologies that both methods employ.
- The PROMETHEE method utilized pairwise comparisons of alternatives based on preference functions to calculate net outranking flows by focusing on how one alternative performed relative to another, considering both the strength and direction of preference. On the other hand, TOPIS adopted a distance-based calculation technique which relied on the geometric distance of each alternative to an ideal (best) and a negative ideal (worst) solution. TOPSIS assumed a linear relationship between criteria and performance, which may lead to different rankings compared to PROMETHEE’s more flexible pairwise comparison.

The current study presented a performance analysis for polymer-modified asphalt (ASA) and polymer–nanocomposite-modified asphalt (ASA-Si) based on different weather conditions and dynamic loading conditions using the experimental results. By employing the MCDA techniques PROMETHEE and TOPSIS, multiple criteria were considered, and analyses were conducted. The experimental results show slight differences between the PROMETHEE and TOPSIS analysis results. Given that the choice of asphalt type in practice depends on various factors, this study aimed to provide decision-makers with insights into the effects of two different modifiers on the asphalt performance under varying weather and loading conditions.

## Figures and Tables

**Table 1:** Specifications of ASA and nanosilica.

Properties	ASA	Nanosilica
**Formula**	ASA (block copolymer)	SiO_2_
**Molecular weight (Da)**	128,000	60.08
**Color and Odor**	White/Odorless	White/Odorless
**Form**	Powder	Nano powder
**Purity**	n/a	0.9999
**Average size**	2 mm	30–50 nm
**Specific gravity**	1.04–1.07	n/a
**Melting point (°C)**	210–240	1600
**Solubility in water**	Insoluble	Insoluble

n/a: not applicable.

**Table 2:** Physical characteristics of the asphalt binders.

Samples	Penetration (mm^−1^)	Softening Point (°C)
Control sample	70	46
3%ASA	48	50
5%ASA	22	56
5%ASA-3%Si	51	53.5
5%ASA-5%Si	37	56.5

## Data Availability

The data are available upon request from the authors.
